# Draft Genome Sequence of *Capniomyces stellatus*, the Obligate Gut Fungal Symbiont of Stonefly

**DOI:** 10.1128/genomeA.00761-16

**Published:** 2016-08-04

**Authors:** Yan Wang, Merlin M. White, Jean-Marc Moncalvo

**Affiliations:** a Department of Ecology and Evolutionary Biology, University of Toronto, Toronto, Ontario, Canada; b Department of Natural History, Royal Ontario Museum, Toronto, Ontario, Canada; c Department of Biological Sciences, Boise State University, Boise, Idaho, USA

## Abstract

*Capniomyces stellatus* is a host-specific endosymbiotic fungus, living in the hindgut of stoneflies (especially in *Allocapnia*). Here, we present the first draft genome sequence of the fungus, as well as the *ab initio* gene prediction and function analyses, which will facilitate the study and comparative analyses of insect-associated fungi.

## GENOME ANNOUNCEMENT

The gut fungus *Capniomyces stellatus*, belonging to Harpellales (Kickxellomycotina), was originally isolated from the hindgut of winter-emerging stonefly nymph, *Allocapnia granulata* (Capniidae) (1). *Capniomyces stellatus* was found strictly associated with the stonefly species and less frequently encountered, except in Arkansas, Missouri, and Tennessee (2, 3). The narrow distribution of the fungus may be due to the inability of long-distance flight of *Allocapnia* spp. and stringent habitat requirements, like the clarity of the water, cool temperature, and special food sources (4).

*Capniomyces stellatus* (MIS-10-108, ARSEF 9258) was obtained from the USDA-ARS Collection of Entomopathogenic Fungal Cultures (ARSEF). Cultures were reproduced using brain heart infusion glucose tryptone medium at room temperature (3). DNA extraction followed the standard CTAB protocol (5). Four PCR-free libraries—500-bp paired-end (PE), 3-kb mate-pair (MP), 5-kb MP, and 10-kb MP—were prepared and sequenced using the Illumina HiSeq 2500 platform (2 × 125-bp read length) at the Donnelly Sequencing Centre, University of Toronto (Toronto, Canada). Raw sequence reads were adapter-trimmed using Trim Galore (http://www.bioinformatics.babraham.ac.uk/projects/trim_galore). Contigs were assembled using Ray version 2.3.1 (6), and scaffolds were built using SSPACE (7). Satellites, simple repeats, and low-complexity sequences were annotated with RepeatMasker version 4.0.5 (http://www.repeatmasker.org) and Tandem Repeat Finder version 4.07b (8), corresponding to fungal sequences from RepBase (9). Gene prediction employed AUGUSTUS version 3.1 (10) using the genome profile of *Conidiobolus coronatus* (Entomophthoramycotina, Zygomycota) (11). Gene functions were annotated as previously described (12), using Blast2GO version 3.2 and InterproScan version 5.8-49.0 (13, 14). TransDecoder (15) was used to predict open reading frames and enable a conservative comparison to estimate gene numbers. CEGMA version 2.4.010312 was used to identify the presence of core eukaryotic protein-coding genes and subsequent evaluation of genome coverage (16). Secreted proteins were predicted using SignalP version 4.1 (17), and transmembrane helices were predicted through the TMHMM server version 2.0 (18).

The presented genome is based on the assembled libraries sequencing data, which amounts to 426 Mb (PE), 1.1 Gb (3- kb MP), 511 Mb (5-kb MP), and 1.3 Gb (10-kb MP), respectively, representing a 130-fold genome coverage on average. The assembly consists of 72 scaffolds, with a total size of 24.8 Mb (*N*_50_, 596 kb). The GC content is 37.8%. We identified 241 out of the 248 eukaryotic core genes. The *ab initio* gene prediction discovered 7,181 genes containing 15,865 exons, as well as 7,831 open reading frames in total. A total of 4.54% of the assembly was identified as low complexity or simple or interspersed repeats, which was masked using RepeatMasker. Functional annotation resulted in gene ontology terms for 3,968 genes and InterPro domains for 5,673 genes, 871 (12.1% of the total) of which were predicted as secreted proteins.

### Nucleotide sequence accession numbers.

This whole-genome project has been deposited in DDBJ/ENA/GenBank under the accession number LUVW00000000. The version described in this paper is the first version, LUVW01000000.
